# A Novel Method for Source Tracking of Chemical Gas Leakage: Outlier Mutation Optimization Algorithm

**DOI:** 10.3390/s22010071

**Published:** 2021-12-23

**Authors:** Zhiyu Xia, Zhengyi Xu, Dan Li, Jianming Wei

**Affiliations:** 1 Shanghai Advanced Research Institute, Chinese Academy of Sciences, Shanghai 201210, China; xiazhiyu2019@sari.ac.cn (Z.X.); lid@sari.ac.cn (D.L.); wjm@sari.ac.cn (J.W.); 2 School of Electronic, Electrical and Communication Engineering, University of Chinese Academy of Sciences, Beijing 100049, China

**Keywords:** Outlier Mutation Optimization algorithm, Gaussian plume model, random walk, emergency response, leakage tracking

## Abstract

Chemical industrial parks, which act as critical infrastructures in many cities, need to be responsive to chemical gas leakage accidents. Once a chemical gas leakage accident occurs, risks of poisoning, fire, and explosion will follow. In order to meet the primary emergency response demands in chemical gas leakage accidents, source tracking technology of chemical gas leakage has been proposed and evolved. This paper proposes a novel method, Outlier Mutation Optimization (OMO) algorithm, aimed to quickly and accurately track the source of chemical gas leakage. The OMO algorithm introduces a random walk exploration mode and, based on Swarm Intelligence (SI), increases the probability of individual mutation. Compared with other optimization algorithms, the OMO algorithm has the advantages of a wider exploration range and more convergence modes. In the algorithm test session, a series of chemical gas leakage accident application examples with random parameters are first assumed based on the Gaussian plume model; next, the qualitative experiments and analysis of the OMO algorithm are conducted, based on the application example. The test results show that the OMO algorithm with default parameters has superior comprehensive performance, including the extremely high average calculation accuracy: the optimal value, which represents the error between the final objective function value obtained by the optimization algorithm and the ideal value, reaches 2.464e-15 when the number of sensors is 16; 2.356e-13 when the number of sensors is 9; and 5.694e-23 when the number of sensors is 4. There is a satisfactory calculation time: 12.743 s/50 times when the number of sensors is 16; 10.304 s/50 times when the number of sensors is 9; and 8.644 s/50 times when the number of sensors is 4. The analysis of the OMO algorithm’s characteristic parameters proves the flexibility and robustness of this method. In addition, compared with other algorithms, the OMO algorithm can obtain an excellent leakage source tracing result in the application examples of 16, 9 and 4 sensors, and the accuracy exceeds the direct search algorithm, evolutionary algorithm, and other swarm intelligence algorithms.

## 1. Introduction

The world’s rapid economic development, urbanization, and industrialization make people increasingly dependent on the chemical industry, machinery industry, and electronics industry, etc. However, the storage of raw materials, the production of spare parts, the assembly of machines, and the testing of technology often need to be carried out in specific places. Among the above industrial processes, the chemical industrial park plays an important role in the storage of raw materials and chemical reaction tests [1,2,3,4]. In chemical industrial parks, there are a large number of chemicals, many of which are flammable, explosive, and hazardous gases [5]. Poisonous gas leakage accidents are challenges for various industries like the petrochemical and gas industries, which will result in financial losses and possibly human injuries [6]. In severe cases, large-scale poisoning disasters or internal domino chain explosion effects may even occur [7].

Toxic gas leakage accidents can be very dangerous to health and the environment. For instance, in December 2018, over 40 workers of a factory in Gujarat’s Valsad district were hospitalized following leakage of chlorine gas; in March 2017, about 450 girl students were hospitalized in southeast Delhi’s Tughlaqabad area following leakage of ‘chloromethyl pyridine’ chemical [8]. Inhaling an excessive amount of toxic gas will cause irreversible harm or even death. Humans have different tolerances for different toxic gases. Table 1 shows the Acute Exposure Guidelines Levels (AEGLs) standard of three kinds of typical toxic gases when supposed the exposure duration is 1 h [9,10,11,12].

The unit of AEGL standard is parts per million (ppm), NR means not recommended due to insufficient data. For the general population, AEGL-1’s airborne concentration will cause notable discomfort but transient and reversible, AEGL-2′s airborne concentration will cause irreversible health effects and even disability, and AEGL-3’s airborne concentration will cause threatening health effects or death [9]. It can be seen from Table 1 that different toxic gases have different concentration threshold standards; even small-scale leak accidents may have irreversible hazards.

To minimize harm to humans and avoid further accidents, it is significant to quickly and accurately get information about the leak point in the chemical leakage accident. Tracking the location of the chemical gas leak source and calculating the average leak rate of the chemical gas leak source are the most important tasks in obtaining the leak point information, which is directly related to the safety and accuracy of the fixed-point elimination work of the emergency safety department [6,13,14]. Using a gas dispersion model to match the actual gas sensor concentration distribution is the mainstream for quickly and accurately getting information about the leak point.

In recent years, many studies have focused on the problem of source tracking of chemical leakage. Singh et al. (2015) used a least-squares approach to inverse modeling, aiming to retrieve multiple point releases in the atmosphere [15,16]. Wang et al. (2018) used a hybrid genetic algorithm to estimate the source of hazardous material releases [17]. Mario et al. (2018) used a Computational Fluid Dynamics (CFD) model to simulate a large amount of gas leak accident data and proposed deep neural network models as a solution to the problem of leak-source tracking [18]. Omar et al. (2020) used conditional bivariate probability function and series signature to realize the spatial identification and temporal prediction of air pollution sources [19]. Huang et al. (2021) compared similarities between source profiles and ambient air profiles, aiming to improve the tracing efficiency [14]. Yet-Pole et al. (2021) combined the benefits of both the CFD model and Gaussian dispersion model, aiming to provide a model basis for the leak of hazardous materials, etc. [4]. The above-mentioned studies mainly include three aspects of such research: the development of the gas dispersion model, the optimization and improvement of the gas dispersion model, and the combination of the gas dispersion model and an external algorithm. Therefore, the gas dispersion model is the basic requirement for the traceability of chemical gas leakage.

There are five mainstream gas dispersion models: Gaussian plume model, Gaussian puff model, Britter and McQuaid (BM) model, Sutton model, and 3-D Finite Element (FEM3) model. The Gaussian plume model and Gaussian puff model are suitable for a continuous leak accident and instantaneous leak accident, respectively. The advantages of Gaussian models are their applicability to various gases, unlimited size of application scenarios, and appropriate model complexity. In addition, Gaussian models’ relatively high consistency between the calculation results and the experimental values make them widely used [4,20,21,22,23,24]. The BM model is based on a series of test data of instantaneous leak and continuous leak of heavy gas, combined by drawing calculation charts; the empirical model has a narrow application range and poor extension [25]. The Sutton model uses the statistical theory of turbulence diffusion to simulate the leak and diffusion process of gas, and there will be large errors [26]. The FEM3 model uses the finite element method to solve the gas leak process-related equations, such as the ideal gas state equation, Navier–Stokes equation, etc. The model has developed rapidly in recent years; the biggest advantage is that it can accurately solve the gas diffusion problem under complex terrain conditions. The shortcomings are detailed meshing, complex solution process, and long calculation time [4,5,27,28,29].

The combination of a gas dispersion model and optimization algorithm has the advantage of various combinations, simple principles, and various applicable scenarios, and is thus widely applied in meeting the demands of source tracking. Table 2 shows the classification and characteristics of some commonly used methods for source tracking of gas leakage.

It can be concluded from Table 2 that considering the economic level, prior technology level and calculation time, the mainstream solution to the problem of gas leakage traceability still relies on the combination of gas dispersion models and optimization algorithms. However, the accuracy of these methods has not reached a high level, which may affect the efficiency of emergency response. For instance, the accuracy of the least-squares method under the one-dimensional gas leakage model is only about 95% [15,16], the accuracy of the DNN under 25 hidden layers is only 75.43% [18], and the approximate errors of PSO, ACO and FA algorithms under the three-dimensional gas leakage models are (Δ*x*, Δ*y*, Δ*Q*) = (43.97%, 49.43%, 19.75%) [30].

In order to trace the source of the gas leak in a satisfactory time and with as high accuracy as possible in chemical gas leakage accidents under flat terrain conditions, this article has made the following innovations:

- Traditional gas leakage accident application examples usually take the position and number of the storage tank or environmental variables as known conditions. In order to illustrate that the OMO algorithm has the best comprehensive performance in the gas leakage traceability problem under flat terrain conditions, randomness is introduced to the various variables that will affect the calculation results of the Gaussian plume model, which can make the application examples created in this article to be strongly representative.
- The introduction of sensor information defect conditions: the number of sensors in the three types of application examples is 16, 9, 4, respectively, and the intensity of the information defect increases in turn, which is a challenge to the exploration integrity of the optimization algorithm, but it is also an important part to prove the advantages of OMO algorithm.
- The OMO algorithm is different from other swarm intelligence algorithms. The exploration phase of the OMO algorithm is dominated by outliers, and the exploration method introduces the Levi flight, which aims to avoid the local optimum with a high degree of exploration. In the exploitation phase of the OMO algorithm, two-way selection improves the convergence speed and accuracy. The complementarity of the encirclement and mutation strategies has become a major feature of the algorithm.
- The research on the parameter law of the OMO algorithm and the introduction of precision control parameters enable the OMO algorithm to adjust parameters reasonably in practical applications and quickly adapt to various application scenarios.

The following content is divided into four sections: methodology, experimental design, results and discussion, conclusions.

## 2. Methodology

### 2.1. Gaussian Plume Model

Last century, the Gaussian model was proposed and applied to the diffusion modeling of actual point sources, including the Gaussian plume model and Gaussian puff model [48]. The Gaussian puff model is suitable for the instantaneous gas leak diffusion from point sources, while the Gaussian plume model is suitable for the continuous gas leak diffusion from point sources [49]. In this paper, the location and intensity of the leak source will be inversely calculated based on the Gaussian plume model.

After the leaking gas is fully mixed with air, the diffusion of the leaking gas is mainly affected by air turbulence, forming passive diffusion. When the turbulent field is stable, the spatial concentration of the leaking gas presents an approximately Gaussian distribution of diffusion. The Gaussian plume model is widely used in the physical movement of gas particles [50,51], and it has been proven to have excellent robustness in the case of small-scale gas point source continuous leak accidents [52]. In contrast, some authors have pointed out that although the Gaussian plume model can better reflect the physical characteristics of gas leak diffusion particles, it cannot handle unpredictable and complex changes, such as a gas leak in extreme climates [53].

Therefore, the Gaussian plume model has the following assumptions [54]:

- The concentration of leaking gas conforms to Gaussian distribution in the *y*-axis section and the *z*-axis section;
- The leakage intensity of the leaking gas is continuous and uniform;
- The ambient wind speed remains constant during the gas leak process, and the default direction is along the positive *x*-axis;
- The model ignores the effects of any chemical reactions, including sedimentation and decomposition;
- The leaked gas follows the ideal gas equation of state and the law of conservation of mass.

When the leak point source is at the origin of the coordinate system, in an infinite space, the expression of the concentration of the leaking gas at a certain position (*x*, *y*, *z*) downstream of the leak source is as follows [55]:

(1) $$C(x,y,z)=\frac{Q}{2\pi u\sigma_{y}\sigma_{z}}e^{-\frac{1}{2}\left(\frac{y^{2}}{\sigma_{y}{}^{2}}+\frac{z^{2}}{\sigma_{z}{}^{2}}\right)}$$

where *C*(*x*, *y*, *z*) is the concentration of the leaking gas at the certain position (*x*, *y*, *z*), unit kg/m^3^; *Q* is the leakage intensity of leak point source (leak rate), unit kg/s; *u* is the average wind speed of the environment, unit m/s; *σ_y_* and *σ_z_* respectively represent the standard deviation of the diffusion of the leaked gas along with the *y* and *z* directions, unit m.

Assuming that the coordinate of the gas leak point source is (*x*_0_, *y*_0_, *h*_0_), and considering the total reflection of the ground surface, the concentration distribution calculation formula of the Gaussian plume model is finally obtained by using the mirror image method:

(2) $$C(x,y,z)=\frac{Q}{2\pi u\sigma_{y}\sigma_{z}}e^{-\frac{{\left(y-y_{0}\right)}^{2}}{2\sigma_{y}{}^{2}}}\left[e^{-\frac{{\left(z-h_{0}\right)}^{2}}{2\sigma_{z}{}^{2}}}+e^{-\frac{{\left(z+h_{0}\right)}^{2}}{2\sigma_{z}{}^{2}}}\right]$$

where all coordinate units in the formula are m.

According to the wind speed and solar radiation intensity, the atmospheric stability can be divided into six different grades A–F, as shown in Table 3 and Table 4 [56,57]. *σ_y_* and *σ_z_* are calculated by the empirical formula of the Pasquill–Gifford model [58], which has a certain correlation with the atmospheric stability level. Their calculation expressions are shown in Table 5 (when the leak source position is at the origin of the coordinates).

### 2.2. Outlier Mutation Optimization (OMO) Algorithm

The OMO algorithm is inspired by animal group hunting behavior and evolutionary algorithms, constructed and improved based on human computing habits. The OMO algorithm aims to overcome the shortcomings of inconsistency between the exploration phase and the exploitation phase of most SI algorithms, and reduce the probability of falling into local optimal solutions, finally improving the accuracy of the solution.

The OMO algorithm is mainly divided into two phases: exploration phase and exploitation phase. The phases are alternated according to the escape energy *E* of the prey:

(3) $$E=2E_{0}\left(1-\frac{t}{T}\right)$$

where *E* is the escape energy of the prey, *E*_0_ ~ U(−1, 1), *t* is the current number of iterations, *T* is the total number of iterations set.

**Figure 1:**
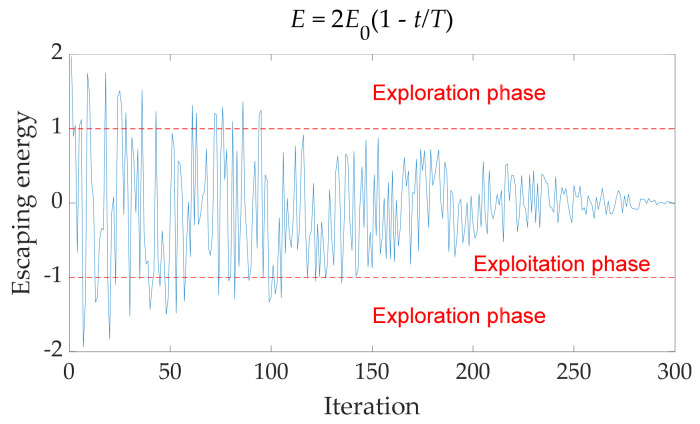
The curve of escape energy *E* varies with iterations.

Figure 1 shows the relationship between the escape energy and the number of iterations in the OMO algorithm. According to the relationship between |*E*| and 1, the OMO algorithm divides the algorithm into the exploration phase and the exploitation phase. To appropriately increase the step length of the exploration phase and prevent excessive jumps in the exploitation phase, a parameter *E_para_* is introduced:

(4) $$E_{para}=2E^{2}$$

In order to adapt to the multi-dimensional exploration interval with different proportions, the step parameters *α* and *β* of Levy flight and Brownian motion are introduced respectively:

(5) $$\alpha =\frac{\left(UB-LB\right)}{k_{\alpha }}S_{H}$$

(6) $$\beta =\frac{\ln\left(UB-LB\right)}{k_{\beta }}S_{R}$$

where *k_α_* and *k_β_* are constants, value 100 in general, *S_H_* is the speed control constant of predator’s Levy flight, which can be adjusted according to the actual situation, default value 1, *S_R_* is the speed control constant of prey’s standard Brownian motion, which can be adjusted according to the actual situation, default value 1, *LB* and *UB* represent the lower limit and upper limit of the exploration area, respectively.

**Figure 2:**
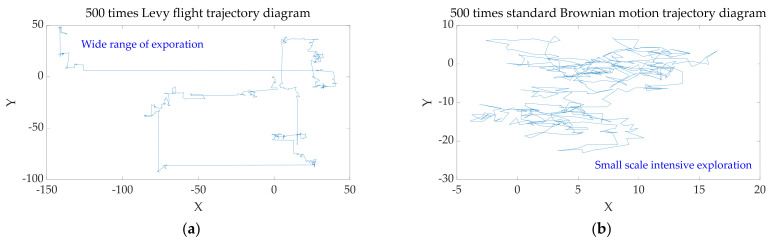
Schematic diagram of two kinds of random walk trajectory, with number of steps = 500. (**a**) Levy flight with the characteristic of wide exploration range; (**b**) Standard Brownian motion with the characteristic of high exploration density.

Standard Brownian motion and Levy flight belong to the Markov process. Figure 2 shows a schematic diagram of the trajectory of 500 steps’ Levy flights and 500 steps’ Brownian motions in a two-dimensional space when the step parameter is 1. Levy flight has the advantage of the wide exploration area, while Brownian motion has the advantages of small space and high density. Combining Levy flight and Brownian motion will enable the OMO algorithm to have a higher degree of exploration and more accurate convergence results.

If the continuous stochastic process {*B*(*t*), *t* ≥ 0} defined in the time domain *t* satisfies the following three properties, it can be called one-dimensional standard Brownian motion (or Wiener process) [59,60]:

- B(0)=0;
- ∀s∈(0,t),B(t)−B(s)~N(0,t−s);
- $\forall s_{1}\in \left(0,t_{1}\right),\forall s_{2}\in \left(0,t_{2}\right),s_{1}\neq s_{2},t_{1}\neq t_{2}$, $B(t_{1})-B(s_{1})$ independent of $B(t_{2})-B(s_{2})$.

*BM* = *B*(*t*) − *B*(*s*) is considered as the single-step length of standard Brownian motion. OMO algorithm takes the default time interval of 1 s, so the single step length of standard Brownian motion *BM* ~ *N*(0, 1).

When |*E*| ≥ 1, predators enter the exploration phase and randomly adopt a strategy to explore:

(7) $$X_{m}(t)=\frac{1}{N}\sum_{i=1}^{N}X_{i}(t)$$

(8) $$X_{i}(t+1)=\;\left\{\begin{array}{ll} \left[X_{rabbit}(t)-X_{m}(t)\right]-r_{1}\left(D\right)\circ \left[r_{2}\left(D\right)\circ \left(UB-LB\right)+LB\right], & q\in [2,3)\\ X_{i}(t)+sign\left[X_{i}(t)-X_{m}(t)\right]\circ abs\left[E_{para}\alpha \circ LF(D)\right], & q\in [1,2)\\ X_{i}(t)+sign\left[X_{i}(t)-X_{rabbit}(t)\right]\circ abs\left[E_{para}\alpha \circ LF(D)\right], & q\in (0,1) \end{array}\right.$$

(9) $$LF=\frac{\mu }{{\left|\nu \right|}^{\frac{1}{\beta }}}$$

(10) $$\mu \sim N(0,\sigma_{\mu }{}^{2})$$

(11) $$\nu \sim N(0,\sigma_{\nu }{}^{2})$$

(12) $$\sigma_{\mu }={\left[\frac{\Gamma \left(1+\beta \right)\sin\left(\frac{\pi \beta }{2}\right)}{\Gamma \left(\frac{1+\beta }{2}\right)\beta 2^{\frac{\beta -1}{2}}}\right]}^{\frac{1}{\beta }}$$

where *X_m_*(*t*) represents the average coordinates of predators in the *t*-generation population, *N* is the total number of individuals in the predators’ population, *X_i_*(*t*) represents the coordinates of the *i*th predator in the *t*-generation population, *X_rabbit_*(*t*) represents the position of the target prey (best fitness) in the *t*-generation population, *LB* and *UB* respectively represent the lower limit and upper limit of the exploration area, the symbol ‘∘’ is the Hadamard product, *D* is the coordinate dimension, *r*_1_(*D*) and *r*_2_(*D*) are *1* × *D*-dimensional vectors that obey (0, 1) uniform distribution, *sign*(*x*) is a sign function, *q* is a constant that obeys (0, 3) uniform distribution, *LF*(*D*) is a 1 × *D*-dimensional step vector of the Mantegna algorithm simulating the Levy flight, Equations (9)–(12) are the expressions of the one-dimensional Mantegna algorithm, generally, *σ_ν_* takes the value 1 and *β* takes the value 1.5.

When *q* ∈ [2, 3), compared with most SI optimization algorithms, the dimension of random parameters is increased, and the dispersion degree of random distribution is improved. When *q* ∈ [1, 2) and *q* ∈ (0, 1), the opposite direction is explored based on the average position of the population and the position of the prey, and *E_para_* appropriately increases the unit step length of the predator individual for Levy flight during the exploration phase, for the purpose of jumping out of the local optimal solution and expanding the scope of exploration in the exploration phase.

When |*E*| < 1, suppose that *r* is the chance of a predator in mutating (*r* ≤ 0.5) or not mutating (*r* > 0.5) before pounce, and then predators enter the exploitation phase:

(13) $$Y=\left\{\begin{array}{ll} X_{rabbit}(t)+E_{para}\left[X_{rabbit}(t)+E_{para}\beta \circ BM(D)-X_{i}(t)\right], & r>0.5\\ X_{r_{1}}(t)+Mu\times \left[X_{r_{2}}(t)-X_{r_{3}}(t)\right], & r\leq 0.5 \end{array}\right.$$

(14) $$Z=\left\{\begin{array}{ll} Y+E_{para}\alpha \circ LF(D), & r>0.5\\ X_{i}(t)\circ H+Y\circ \sim H, & r\leq 0.5 \end{array}\right.$$

(15) $$H\left(1,i\right)=\left\{\begin{array}{l} 1,\;rand\left(0,1\right)>Cr\\ 0,\;rand\left(0,1\right)\leq Cr \end{array}\right.,i=1,2,\ldots ,D$$

(16) $$F_{\min}=\min\left\{F[X_{i}(t)],F(Y),F(Z)\right\}$$

(17) $$X_{i}(t+1)=\left\{\begin{array}{l} X_{i}(t),\\ Y,\\ Z, \end{array}\right.\begin{array}{l} F_{\min}=F[X_{i}(t)]\\ F_{\min}=F(Y)\\ F_{\min}=F(Z) \end{array}$$

where *BM*(*D*) is a *1 × D*-dimensional step vector of the standard Brownian motion, *r* is a constant that obeys (0, 1) uniform distribution, *r*_1_, *r*_2_, *r*_3_ are random integers between [1, *N*] and are not equal to each other, *Mu* is the coefficient of variation, values between [0.4, 1], *H* is a 1 *× D*-dimensional logical vector aimed to increase the diversity of genes in the population, *Cr* ∈ [0, 1], which means the crossover probability of the individual’s genes, and the *F* function is the objective optimization function.

The exploitation phase in the OMO algorithm is divided into two strategies: besiege strategy and mutant strategy. In besiege strategy, the method of combining the small-scale Brownian motion of prey and the shorter step length Levy flight of predators is adopted, which can not only jump out of the local optimal solution but also conduct sufficient small-scale exploration around the prey for high convergence accuracy of the algorithm. In the mutant strategy, predators take mutation and crossover operations; this strategy absorbs the advantages of the integration of exploration and convergence in evolutionary algorithms, guarantees the diversity of the population in the early exploration, and avoids the shortcomings of decreased exploration ability after the exploitation phase is transferred to the exploration phase.

**Figure 3:**
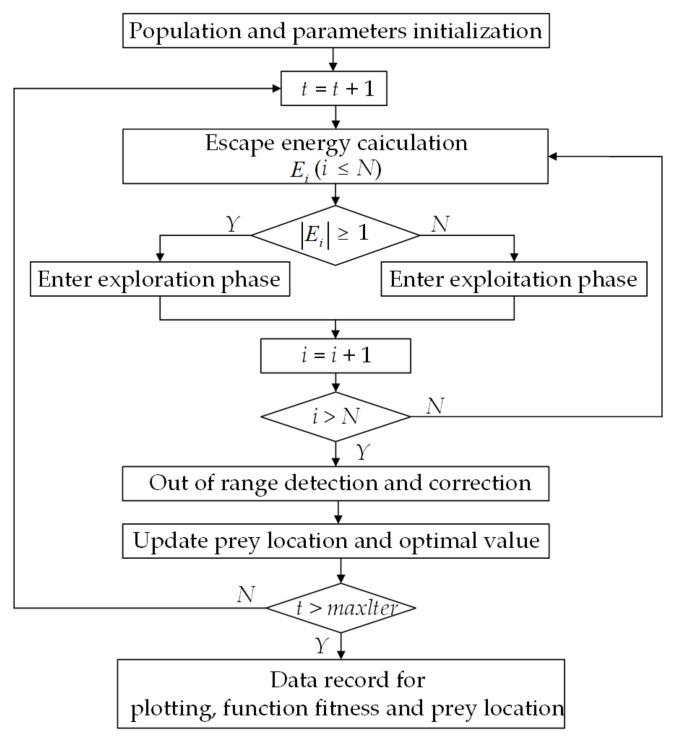
Flow chart of the OMO algorithm.

In order to better show the logic of the OMO algorithm, Figure 3 shows a brief flow chart of the OMO algorithm.

The OMO algorithm has superior performance in most standard test functions. Table 6 shows the details of the six representative standard test functions [61,62]. The population number *N* and maximum iterations of the OMO optimizer are set to 30 and 300, respectively. The speed control constant of the predator’s Levy flight *S_H_* is set to default value 1, and the speed control constant of prey’s standard Brownian motion *S_R_* is also set to default value 1, other parameters are consistent with those given in Table 6.

**Figure 4:**
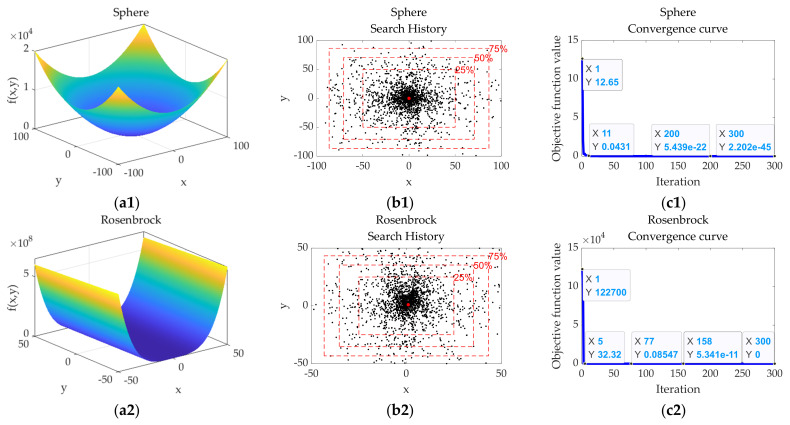

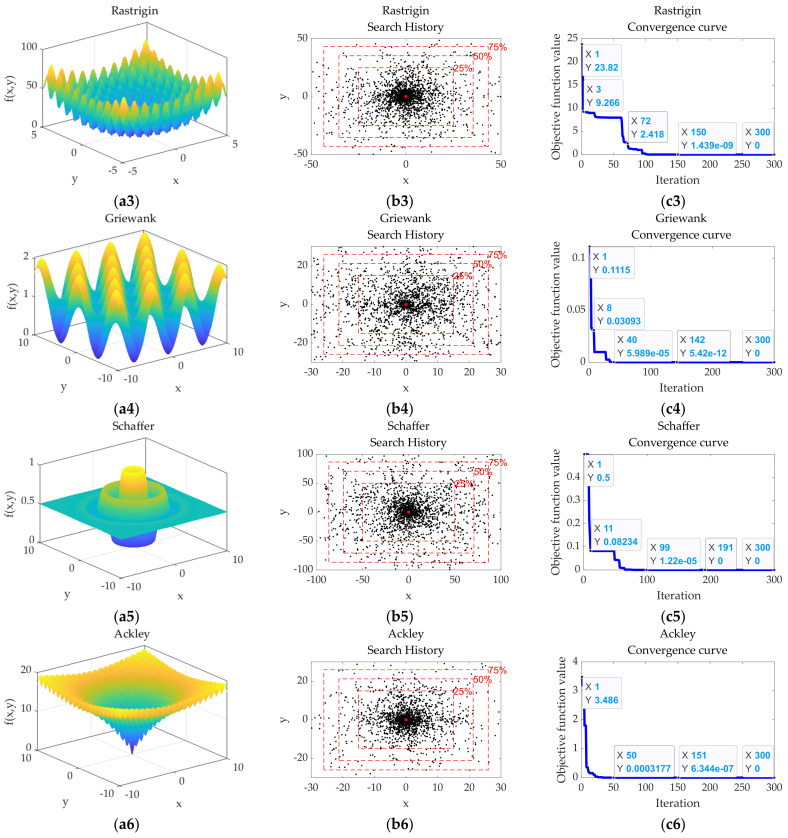
The standard test results of the OMO algorithm when the dimension is 2. (**a1**–**a6**) Parameter space schematic diagram of the *i*th test function; (**b1**–**b6**) Search history diagram of the OMO algorithm in the *i*th test function with percentage range mark; (**c1**–**c6**) The optimal value of the function varies with the number of iterations graph of the OMO algorithm in the *i*th test function.

Figure 4 shows the test results of the OMO algorithm when the dimension is 2. The equations and parameter range of the test functions are consistent with those shown in Table 6. The population number *N* and the number of iterations of the OMO algorithm are set to 30 and 300, respectively.

The test results of the OMO algorithm are shown in Figure 4. The search history diagram reflects the characteristics of sufficient peripheral exploration degree, high randomness of exploration, and strong convergence ability in the OMO algorithm. The convergence curve reflects the advantage of the OMO algorithm that can effectively jump out of the local optimal solution.

The experiment of chemical gas leakage’ source tracking will be detailed in Section 3 and result details of the OMO algorithm will be explained in Section 4.

## 3. Experimental Design

For the chemical gas leakage accident under flat terrain, this paper proposes the combination of the OMO algorithm and Gaussian plume model to trace the source of chemical gas leakage. The experiment is mainly to verify the accuracy and efficiency of the OMO algorithm in solving this kind of problem, the influence of different OMO algorithm parameters on the results, and the advantages compared with other optimization algorithms.

### 3.1. Chemical Gas Leakage Accident Application Example

This paper proposes a novel algorithm to quickly and accurately realize the source tracking of chemical gas leakage. It is, therefore, necessary to first establish a chemical gas leakage accident application example in order to verify the application effect of the OMO algorithm.

A single application example cannot prove the universality of the OMO algorithm in practical application scenarios. However, more application examples and respective tests also cannot fully cover all the possibilities. Therefore, the category characteristics of the chemical gas leakage accident scene with flat terrain and stable atmospheric conditions need to be fixed, and the variables need to increase randomness. Experiments based on this condition can strongly prove that the OMO algorithm can be efficiently applied to the chemical gas leakage accident scene with flat terrain and stable atmospheric conditions.

In the chemical gas leakage accident scene with flat terrain and stable atmospheric conditions, the main variables include scene range, the location, and intensity of the gas leakage source, average ambient wind speed, atmospheric stability, and the location distribution of gas sensors. Because the entire gas leakage scene can be scaled proportionally, so all application examples belonging to the scene mentioned above can be equivalent to the application examples where the scope of the scene is fixed at 100 × 100 and other variables are random. The atmospheric stability rank has a clear classification and calculation formula, which has little influence on the optimization algorithm and indirectly affects the value of the gas sensors, so this paper does not increase the randomness of the atmospheric stability. Considering the reliability of experimental results and the algorithm evaluation system, multiple groups of independent small-scale tests will be conducted in each experiment. All the obtained optimal values, location values of the gas leakage source and intensity values of the gas leakage source will be averaged respectively, and the results after averaging need to have definite comparison indexes. Therefore, each experiment will keep the intensity and location of the gas leakage source unchanged and add the randomness of the gas sensors distribution and the randomness of the average ambient wind speed to the application example. In addition, although different gas leakage source locations and intensities will form different objective functions, the formed objective functions belong to the same type and have little impact on the comprehensive performance evaluation of the optimization algorithm. Therefore, the test results in this paper only show and analyze one of the representative cases.

**Figure 5:**
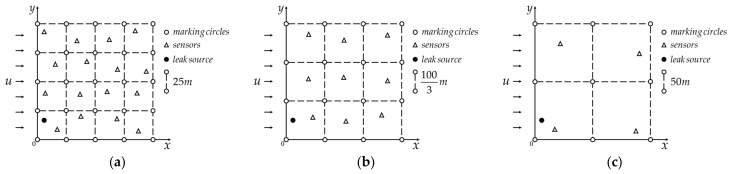
Schematic diagram of three application examples. (**a**) Distribution of 16 monitoring points and leak source; (**b**) distribution of 9 monitoring points and leak source; (**c**) distribution of 4 monitoring points and leak source.

Suppose that in a flat terrain chemical industrial park with a range of 100 m × 100 m, a chlorine storage tank at an unknown location has a leakage accident. The average leakage rate (the leakage intensity) is *Q* = 80 kg/s, the location of the leakage source is (*x*_0_, *y*_0_, *h*_0_) = (8, 15, 1) m, atmospheric stability level rank D, the average environmental wind speed is *u* = (10 × *r_w_*, 0, 0) m/s, and the plane distribution of the gas concentration sensors are as shown in Figure 5, with their height assumed to be 0 m and a measurement unit of kg/m^3^. Supplementary notes: *r_w_* obeys the uniform random distribution between (0, 1), the 16, 9, 4 sensors are randomly distributed in 4 × 4, 3 × 3, 2 × 2 squares formed by hollow circles, respectively. The accuracy of an ordinary commercial chlorine sensor (e.g., ME3-CL2) is 0.1 ppm, which is approximately equal to 3.155e-8 kg/m^3^. For the convenience of calculation, the default sensor accuracy is set to 1.0e-8 kg/m^3^ in this article.

### 3.2. Chemical Gas Leakage’s Source Tracking Model

In order to simplify the chemical gas leakage’s source tracking model, this paper ideally considered that the chlorine storage tanks in the chemical industrial park are made following the Chinese industrial standard of 1 m height. Therefore, when a leakage accident occurs, the parameters that need to be back-calculated are the top view plane position (*x*_0_, *y*_0_) of the leakage source and the average leakage rate *Q* of the leakage source.

The construction of the chemical gas leakage’s source tracking model is based on the Gaussian plume model. Suppose the total number of deployed sensors is *NS*, the chlorine concentration observation value obtained by deployed sensors is *C_ob_*(*i*), and the theoretical value calculated by the diffusion model is *C_cal_*(*i*), where *i* is the serial number of the sensors. Then the objective function can be expressed in the form of Equation (18):

(18) $$\underset{\left(x_{0},y_{0},Q\right)}{\min}f\left(x_{0},y_{0},Q\right)=\sum_{i=1}^{NS}{\left(C_{ob}\left(i\right)-C_{cal}\left(i\right)\right)}^{2}$$

Suppose the position of the *i*th sensor is (*x_i_*, *y_i_*, *z_i_*), then the theoretical value calculated by the Gaussian plume diffusion model can be expressed in the form of Equation (19):

(19) $$C_{cal}\left(i\right)=\frac{Q}{2\pi u\sigma_{y_{i}}\sigma_{z_{i}}}e^{-\frac{{\left(y_{i}-y_{0}\right)}^{2}}{2\sigma_{y_{i}}{}^{2}}}\left[e^{-\frac{{\left(z_{i}-h_{0}\right)}^{2}}{2\sigma_{z_{i}}{}^{2}}}+e^{-\frac{{\left(z_{i}+h_{0}\right)}^{2}}{2\sigma_{z_{i}}{}^{2}}}\right]$$

According to Table 5, the Pasquill–Gifford diffusion coefficient equation of atmospheric stability level D at the position of the *i*th sensor is obtained:

(20) $$\sigma_{y}=0.16\left(x_{i}-x_{0}\right){\left[1+0.0004\left(x_{i}-x_{0}\right)\right]}^{-1/2}$$

(21) $$\sigma_{z}=0.14\left(x_{i}-x_{0}\right){\left[1+0.0003\left(x_{i}-x_{0}\right)\right]}^{-1/2}$$

Based on the chemical gas leakage accident application example and chemical gas leakage’s source tracking model, the total number *NS* of deployed sensors is 16, 9, 4, respectively, the lower limit of the optimization interval is *LB* = (0, 0, 0) and the upper limit is *UB* = (100, 100, 200), and the concentration observation values of monitoring points are simulated according to the Gaussian plume model.

In addition, the total number of sensors is set to 16, 9, 4 because the economic factors of sensor purchasing and placing in actual chemical industrial parks are taken into account, and monitoring points’ data with insufficient information are more likely to lead to an increase in the number of locally optimal solutions, thus reflecting the advantages of each optimization algorithm.

### 3.3. Other Details and Pseudocode of OMO Algorithm

All algorithms were implemented under MathWorks MATLAB R2020a (MathWorks.Inc, Natick, MA, USA) on a computer with a Windows 10-64bit professional edition (Microsoft, Redmond, WA, USA), 16GB RAM, and Intel^®^ Core™ i5-9400F. The population number *N* and maximum iterations of all optimizers are set to 30 and 300, respectively. The geometric mean has the advantage of good stability, especially when analyzing the value of the objective function; all results are recorded and calculated based on the geometric mean of optimizers over 50 independent runs. Each independent operation will reset the random variables in the application example to achieve a high degree of randomness in the application example, which aims to comprehensively evaluate the multi-scenario application capabilities of each algorithm. The OMO algorithm’s parameters use default value and will be tested in Section 4, and the parameters of other algorithms are the same as the default or recommended value, which are reported in Table 7.

The pseudocode of the proposed OMO algorithm is reported in Algorithm 1.
**Algorithm 1.** Pseudocode of OMO algorithm.**Inputs:** The population size *N*, maximum number of iterations *T*, Speed Control Constant *S_H_*, *S_R_* **Outputs:** Data required for drawing *plotData*, the location of prey *preyLocation* and its fitness value *preyEnergy*Initialize the random population *X_i_*(*i* = 1, 2, …, *N*), *plotData*, *preyLocation*, *preyEnergy* and step parameters *α*, *β* **for** (each iteration *t*) **do**  **for** (each predator *X_i_*) **do**    Update the escape energy *E* and *E_para_* using Equations (3) and (4)    **if** (|*E*| ≥ 1) **then**                   ⊳Exploration phase      Update the location vector using Equation (8)    **if** (|*E*| < 1) **then**                   ⊳Exploitation phase      **if** (*r* > 0.5) **then**                   ⊳Besiege strategy        Update the location vector using Equations (13), (14), (16), (17)        **if** (*r ≤* 0.5) **then**                 ⊳Mutant strategy        Update the location vector using Equations (13)–(17)  Checkup population boundary and correct individual  Calculate the fitness values of predator population  Set *preyLocation* and *preyEnergy* as the location of prey and its fitness based on the minimum value between last prey and current population  Update *plotData* and record them **Return** *plotData*, *preyLocation*, *preyEnergy*

## 4. Results and Discussion

### 4.1. Results

Please note that the calculation time of all algorithms in this section is obtained by summing after 50 independent runs. For experiments that are not affected by the number of sensors, this paper will only show the test results under the 16 sensor scenarios.

#### 4.1.1. Qualitative Results of OMO Algorithm

Table 8, Table 9 and Table 10 reveal the qualitative results of the OMO algorithm applied to the application example constructed above. It can be seen that when the number of sensors is 16 and 9, the OMO algorithm shows extremely high accuracy in the problem of chemical gas leakage traceability, and when the number of sensors is 4, that is, when the data is severely insufficient, the OMO algorithm will have some deviation, but there is still a certain degree of accuracy.

**Figure 6:**
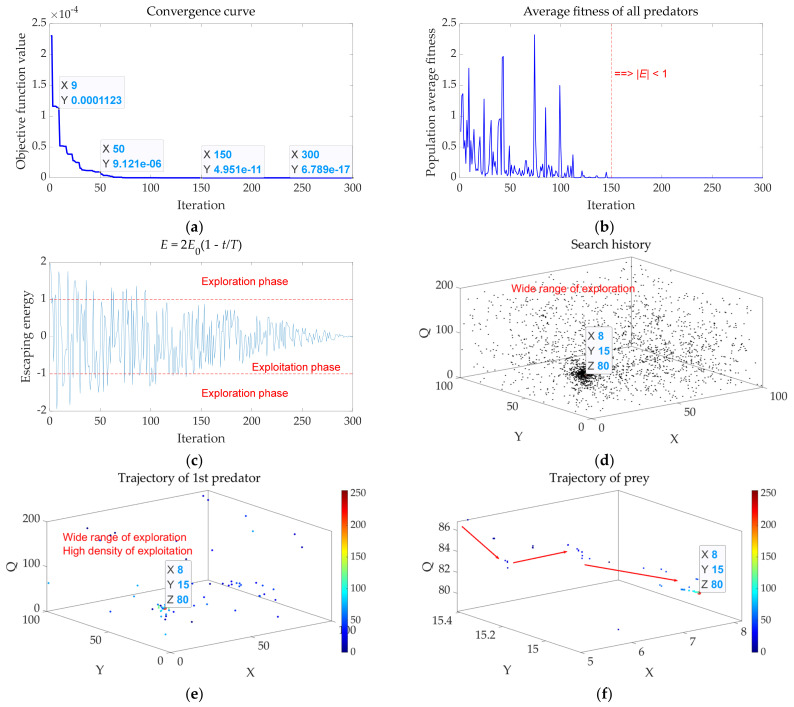
Qualitative results figures of OMO algorithm under 16 sensors. (**a**) Convergence curve; (**b**) average fitness of all predators; (**c**) escape energy of the prey; (**d**) search history of predators; (**e**) trajectory of the first predator; (**f**) trajectory of the prey.

The qualitative results figures of the OMO algorithm applied to the chemical gas leakage accident application example constructed above are demonstrated in Figure 6. These results include five well-known metrics: convergence behavior, the average fitness of the population, search history, the trajectory of the first predator, and the trajectory of the prey. In addition, the escape energy of the prey is also monitored during iterations. The convergence curve reveals how the optimal value of the objective function varies during optimization. The average fitness of the population monitors how the average fitness of whole predators varies during the process of optimization. The search history distribution diagram shows the history of those positions visited by artificial predators during the calculation. The trajectory of the first predator diagram shows how the first variable of the first predator individual varies during the calculation. The map of the trajectory of the prey monitors how the variable of the prey (best solution’s location) varies during the iterations.

From the search history of predators, it can be concluded that predators attempt to initially boost the diversification and explore the easily overlooked areas of solution space and then exploit the vicinity of the best locations. The wide exploration area can better reduce the search omissions.

As in the trajectory in Figure 6, we see that the foremost predator’s amplitude of these variations covers more than 50% of the solution space while the prey’s amplitude of these variations covers about 25% of the solution space. As time passes, the explores amplitudes of the foremost predator and the prey gradually decrease. This point guarantees the transition of the OMO algorithm from the exploratory phase to the exploitative phase. Eventually, the motion patterns of the first predator and the prey become very stable which shows that the OMO algorithm is exploiting the promising regions during the concluding steps.

From the fitness and average fitness curve, it can be observed that the OMO algorithm jumped out of the local optimal solution multiple and reached the global optimal solution about the 50th iteration, but the convergence of the average fitness is reached at about 150th iteration, which means that the OMO algorithm applies the greedy algorithm which is not satisfied with the optimal solution in the previous iteration and tries to jump out of the local optimal solution. Thus, when facing the problem of location optimization with a known optimal value of the objective function, a precision control constant can be introduced to realize the function of stopping iteration after the required precision is reached, thereby greatly shortening the calculation time. The test results of the chemical gas leakage accident application example are shown in Table 11 above.

**Figure 7:**
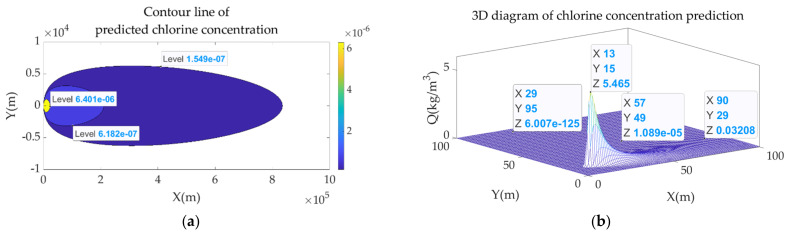
Supplement to chemical gas leakage accident system. (**a**) Prediction of gas concentration distribution and contour division under the AEGLs standard; (**b**) 3D diagram of prediction of gas concentration distribution.

Although the OMO method is mainly used to solve the traceability problem of chemical gas leakage, for the entire chemical gas leakage accident emergency and response system, the prediction of gas concentration and the marking of dangerous thresholds are also an important part of it. Figure 7 shows a preliminary schematic diagram of gas concentration prediction and the mark of risk thresholds.

#### 4.1.2. Relationship between OMO Algorithm and Population Number *N*

**Figure 8:**
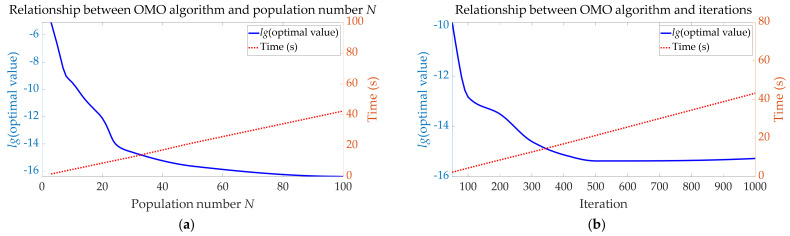
Relationship between OMO algorithm and population number *N* or iterations. (**a**) Smooth curve graph of the population number *N*—the optimal value of the objective function and the population number *N*—the consuming time; (**b**) smooth curve graph of the iterations—the optimal value of the objective function and the iterations—the consuming time.

In the case of a fixed number of 300 iterations, the results of the relationship between the OMO algorithm and population number *N* are shown in Table 12 and Figure 8a.

Taking the Briggs logarithm of the optimal value as the criterion for judging the accuracy of the OMO algorithm, the conclusions can be drawn from Table 12 and Figure 8a:

- The time-requirement of the OMO algorithm is approximately proportional to the population number *N*;
- The accuracy of the OMO algorithm gradually increases as the population number *N* increases;
- When the population number 3 < *N* < 5, the accuracy of the OMO algorithm is relatively low; when the population number 3 < *N* < 25, the accuracy of the OMO algorithm is significantly improved with the increase of *N*; when the population number *N* > 25, the increased speed of OMO algorithm’s accuracy with the increase of *N* gradually decreases and stabilizes.

Figure 8 combines the results of Section 4.1.2 and Section 4.1.3. It can be concluded from Figure 8 that the population size *N* and the number of iterations have similar effects on the OMO algorithm in terms of time-requirements and accuracy.

#### 4.1.3. Relationship between OMO Algorithm and Iterations

In the case of a fixed number of *N* = 30, the results of the relationship between the OMO algorithm and iterations are shown in Table 13 and Figure 8b.

Taking the Briggs logarithm of the optimal value as the criterion for judging the accuracy of the OMO algorithm, the conclusions can be drawn from Table 13 and Figure 8b:

- The time-requirement of the OMO algorithm is approximately proportional to the iterations;
- The accuracy of the OMO algorithm gradually increases as the iterations increase;
- When the iterations <100, the accuracy of the OMO algorithm is significantly improved with the increase of iterations; when the 100 < iterations < 500, the accuracy of the OMO algorithm is slightly improved with the increase of iterations; when the iterations >500, the increased speed of the OMO algorithm’s accuracy with the increase of iterations gradually decreases and stabilizes.

#### 4.1.4. Relationship between OMO Algorithm and Speed Control Constant

**Figure 9:**
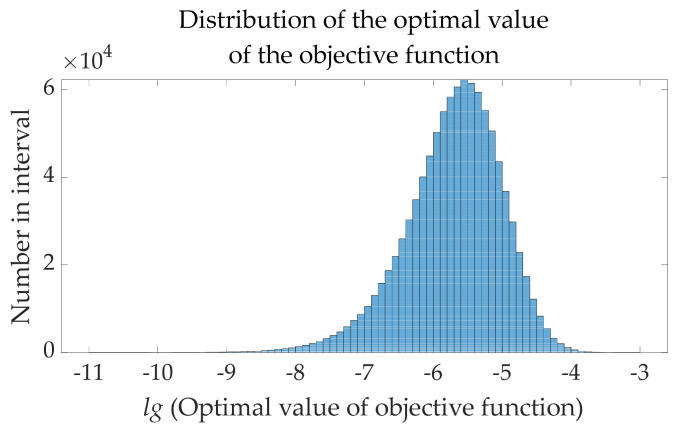
Experiment data set of the optimal value of the objective function calculated by OMO algorithm distribution statistics histogram.

**Figure 10:**
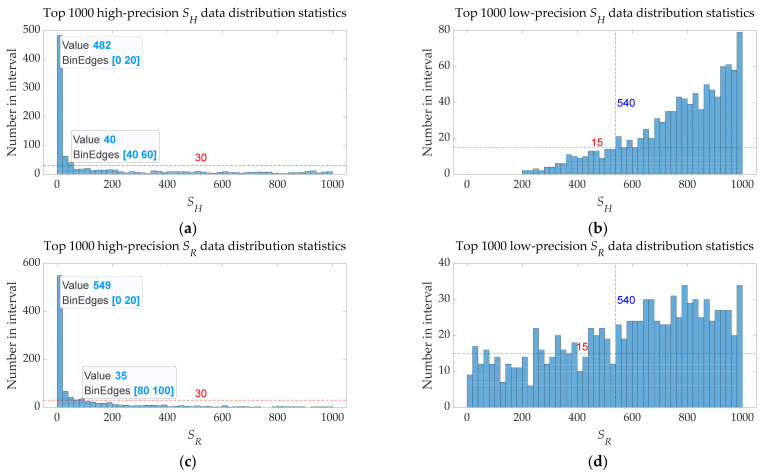
Speed control constant—OMO algorithm’s accuracy distribution histogram. (**a**) *S_H_* distribution histogram of the first 1000 sets of high-precision data; (**b**) *S_H_* distribution histogram of the first 1000 sets of low-precision data; (**c**) *S_R_* distribution histogram of the first 1000 sets of high-precision data; (**d**) *S_R_* distribution histogram of the first 1000 sets of low-precision data.

Given the high number of iterations and the high population number, the results calculated by the OMO algorithm will show high accuracy, which is not inefficient in studying the relationship between the OMO algorithm and speed control constant. Therefore, in the case of fixed numbers of *r* = 0, 100 iterations, and 20 population numbers, 1,000,000 sets of data are recorded based on the free combination of *S_H_* = 1:1:1000 and *S_R_* = 1:1:1000. The results of the relationship between the OMO algorithm and speed control constant are shown in Table 14, Figure 9 and Figure 10.

These conclusions can be drawn from Table 14, Figure 9 and Figure 10:

- Changing the values of *S_H_* and *S_R_* will have a certain impact on the accuracy of the OMO algorithm, but random values of *S_H_* and *S_R_* will have a high probability to get a more satisfactory result.
- High-precision OMO algorithm results are often accompanied by suitable exploration steps (influenced by *S_H_*), and low-precision OMO algorithm results are often accompanied by too-large exploration steps. The reason why some *S_H_* are too large but can also achieve accuracy of the algorithm is mainly due to the random allocation of super boundary data, but in the chemical gas leakage accident model established by this paper, the best value of *S_H_* is 1–60.
- High-precision OMO algorithm results are often accompanied by suitable exploitation steps (influenced by *S_R_*), and low-precision OMO algorithm results are often accompanied by too large exploitation steps. Logarithmic processing has been performed in the step length parameter of the standard Brownian motion to ensure that the exploitation space is small enough in the final stage and the space has been explored completely; therefore, a small-range change of *S_R_* will have little effect on the accuracy of the OMO algorithm. In the chemical gas leakage accident model established by this paper, the best value of *S_R_* is 1–100.

Therefore, the default values of the speed control constant for the chemical gas leakage accident model (16 sensors) established by this paper can be decided: *S_H_* = 4 and *S_R_* = 6. The optimal speed control constant values of different application examples are not necessarily the same and need to be verified by experiments, but most of the speed control constant settings can get a satisfactory result with a high probability.

#### 4.1.5. Robustness Analysis under the Influence of White Gaussian Noise (WGN)

Considering the actual factors of the chemical gas leakage accident application example, we can add White Gaussian Noise (WGN) with different Signal-to-Noise Ratios (SNRs) to the observed value of the gas concentration to simulate the error between the calculated value of the Gaussian plume model and the actual observed value, and analyze the robustness of the OMO algorithm. In the case of fixed numbers of *N* = 30, iterations = 300, *S_H_* = 4, and *S_R_* = 6, the results of the relationship between the OMO algorithm’s accuracy and SNR are shown in Table 15.

From the data in Table 15, it can be found that as the noise increases, the error of the calculation result increases relatively. When SNR > 15, the deviation of the leakage source data calculated by the OMO algorithm is within the allowable range of error, but when SNR < 1, the results do not meet the ideal expectations.

In summary, the OMO algorithm has great robustness and can be congruently applied to chemical gas leakage accident scenarios whose SNR > 15.

#### 4.1.6. Comparative Analysis among Optimization Algorithms

In the case of fixed numbers of *N* = 30, iterations = 300, *S_H_* = 1, and *S_R_* = 1 in the OMO algorithm, the comparison results of various optimization algorithms are shown in Table 16, Table 17 and Table 18. In addition, all optimization algorithms were implemented based on the application example established in Section 3.1. The population number *N* of other optimizers is set to 30, except for the PS and SA algorithms, which do not have the parameter of population number *N*, and the number of iterations of other optimizers is set to 300. The basic parameters of other algorithms are the same as the default or recommended value, which are reported in Section 3.3, Table 7.

The PSO algorithm has the fastest calculation speed: 2.223 s/50 times. The OMO algorithm has the lowest geometric mean error: Δ*x*_0_ = 1.971%, Δ*y*_0_ = 0.131%, Δ*Q* = 1.193%, and the stable highest accuracy, which can be reflected by the geometric mean of the optimal value: 2.464e-15.

The PSO algorithm has the fastest calculation speed: 1.638 s/50 times. The lowest *x*_0_ error, the lowest *y*_0_ error, and the lowest *Q* error are: Δ*x*_0_ = 3.593% calculated by OMO, Δ*y*_0_ = 0.010% calculated by DE, Δ*Q* = 0.077% calculated by GA. The OMO algorithm has the stable highest accuracy: 2.356e-13.

The PSO algorithm has the fastest calculation speed: 1.341 s/50 times. The lowest *x*_0_ error, the lowest *y*_0_ error, and the lowest *Q* error are: Δ*x*_0_ = 23.427% calculated by GA, Δ*y*_0_ = 1.121% calculated by SA, Δ*Q* = 0.709% calculated by GWO. The OMO algorithm has the stable highest accuracy: 5.694e-23.

Analyzing and comparing the results in Table 11 and Table 16, it can be concluded that the calculation speed of PS, SA, PSO, and GA algorithms are significantly better than other algorithms, but the calculation accuracy of PS, SA, and GA algorithms is significantly lower than most algorithms, while the calculation accuracy of PSO algorithms is relatively higher. The DE algorithm has great advantages in calculation time and calculation accuracy, swarm intelligence algorithms such as HHO, GWO, SSA, WOA performance is mediocre in the calculation speed aspects, and the SSA algorithm surpasses other algorithms in accuracy, second only to the OMO and DE algorithms. The OMO algorithm has the highest calculation accuracy among all algorithms, at the expense of calculation time. However, the OMO algorithm, which introduces precision control parameters, achieves a balance between precision and calculation, when the optimal value of the function reaches 1e-10 (only second to the DE algorithm) and the calculation time is only about 6.697s (only second to PSO, GA algorithms, and PS, SA algorithms which belong to the direct search algorithm category, which is known for its computational speed).

Analyzing and comparing the results in Table 16, Table 17 and Table 18, it can be concluded that regardless of the severity of sensor data insufficiency in the chemical gas leakage scene, the OMO algorithm always maintains great advantages in accuracy compared with other algorithms. The geometric average of the optimal value mainly reflects the average accuracy of the algorithm; that is, the stability and accuracy of the OMO algorithm are higher than other optimization algorithms. When the number of sensors is reduced from 16 to 9, the accuracy of some optimization algorithms is severely reduced, mainly because the lack of information leads to an increase in the local optimal value. When the number of sensors is 9, optimization algorithms that perform better include OMO, PSO, DE, HHO and SSA. When the number of sensors is only 4, the severe data insufficiency leads to dense local optimal values, and each optimization algorithm fails to obtain relatively accurate results. However, from the perspective of the relative error and the average optimal value, the OMO algorithm is more suitable than others for solving the source tracking of chemical gas leakage problems.

In summary, the OMO algorithm has shown extremely high accuracy when facing the application example of chemical gas leakage source tracking. The OMO algorithm introduces precision control parameters that can provide high-precision results while ensuring the calculation speed, thereby meeting the emergency needs in the event of chemical gas leakage accidents.

### 4.2. Discussion

As per results in previous sections, we can recognize that the OMO algorithm shows significantly superior results for the application example of source tracking of chemical gas leakage.

The following experiments, studies, or analyses on the OMO algorithm have been described in this Section: qualitative results of the OMO algorithm in attempting to solve the application example proposed in Section 3, relationship between OMO algorithm and population number *N*, relationship between OMO algorithm and iterations, relationship between OMO algorithm and speed control constant, robustness analysis under the influence of WGN and comparative analysis among optimization algorithms. The following conclusions or default values can be obtained from these experiments, studies, and analysis: the OMO algorithm has excellent exploration and exploitation capabilities; the larger the number of populations, the higher the accuracy of the OMO algorithm; the larger the iterations, the higher the accuracy of the OMO algorithm; the speed control constant has a certain degree of influence on the accuracy of the OMO algorithm, default *S_H_* = 4 and *S_R_* = 6; the OMO algorithm has relatively good robustness and can calculate reasonable results when SNR > 15 in solving the application example; the accuracy of the OMO algorithm is far superior to other algorithms, and the OMO algorithm that introduces precision control parameters is better than other algorithms in terms of overall performance.

In future work, the basic emergency safety system can be established. Real-time prediction of chemical gas dispersion will be added into this system and the Gaussian plume model will be replaced by a higher precision model with the guarantee of viable calculation time-requirements. First, we will conduct more experiments based on the CFD model and establish a database as reference data for real chemical leakage accidents. Second, the chemical gas leakage scenario experiments will be conducted to verify the established database. Third, we will use deep learning or other feasible methods aimed to establish a universal gas diffusion empirical model. Finally, we will test the accuracy of the model and apply it to the prediction of gas diffusion and replace the Gaussian plume model with the OMO algorithm.

## 5. Conclusions

This paper introduces a novel method for the purpose of chemical gas leakage accident source tracking. The OMO algorithm is mainly divided into two phases. In the exploration phase, Levi’s flight and its step size vector are introduced, and some individual outlier search is adopted to improve the search range at the initial phase of the exploration. In the exploitation phase, Brownian motion and its step size vector are introduced, and the idea of individual mutation is also introduced, which enriches the path of convergence, and improves the convergence speed and accuracy. As regards the computational efficiency of the OMO algorithm, the test results show that the OMO algorithm with default parameters has superior comprehensive performance, including the extremely high average calculation accuracy—the optimal value reaches 2.464e-15 when the number of sensors is 16; 2.356e-13 when the number of sensors is 9; and 5.694e-23 when the number of sensors is 4. There is a satisfactory calculation time: 12.743 s/50 times when the number of sensors is 16; 10.304 s/50 times when the number of sensors is 9; and 8.644 s/50 times when the number of sensors is 4.

In recent years, the safety of chemical industrial parks has received great attention, especially in China. In order to actively respond to chemical gas leakage accidents, a complete set of safety emergency systems needs to be rigorously developed, including the source tracking of chemical gas leakage accidents and the real-time prediction of chemical gas dispersion. In addition to these basic emergency needs, there are challenges of source tracking of chemical gas leakage accidents with complex terrain and climatic conditions, the real-time predicting of fire and explosion caused by leaking gas, etc.

The OMO algorithm combined with a Gaussian plume model is proved in this paper to be able to quickly and accurately realize the inverse calculation of gas leakage sources under flat terrain and stable atmospheric conditions. This method can provide technical support for emergency measures in normal chemical industrial parks, and can also provide some help for the future study of chemical gas leakage accidents under complex situations.

The OMO algorithm can not only be applied to chemical gas leakage accidents in chemical industrial parks but also the three-bar truss design problem, tension/compression spring design problem, gas pipeline leakage accidents, pressure vessel design problem, etc. Conclusions can be drawn from the results of Section 4: OMO’s flexible algorithm parameters can improve its effect on different application examples to a certain extent, including speed control constant, precision control coefficient, the maximum number of iterations, and population number.

## Figures and Tables

**Table 1:** Ammonia, chlorine, and phosgene, 1 h exposure duration. Results—AEGL Program.

	Ammonia	Chlorine	Phosgene
AEGL-1	30	0.5	NR
AEGL-2	160	2.0	0.3
AEGL-3	1100	20	0.75

**Table 2:** Methods for source tracking of chemical gas leakage.

Algorithm Classification	Strengths	Weaknesses	Instances
Swarm Intelligence (SI) algorithms based on gas dispersion models	High degree of freedom in the exploration phase, fast calculation speed, and accurate result in the exploitation phase	Insufficient random exploration may make the result fall into local optimal solution	Particle Swarm Optimization (PSO) Algorithm [30,31] Ant Colony Optimization (ACO) Algorithm [30] Firefly Algorithm [30] Harris Hawks Optimization (HHO) Algorithm [32]
Evolutionary algorithms based on gas dispersion models	No need to distinguish the exploration and exploitation phases; controllable search ability (coefficient of variation)	High evolutionary generations will reduce population diversity	Genetic Algorithm (GA) [17,33] Differential Evolution (DE) Algorithm [34]
Direct optimization algorithms based on gas dispersion models	Low algorithm complexity and fast calculation speed	Easy to fall into local optimal solution	Pattern Search (PS) Algorithm [35,36] Simulated Annealing (SA) Algorithm [37,38,39] Least Squares Algorithm [15,16]
Methods based on big data or probabilistic analysis	No need to build a scene model, not restricted by geographical conditions	Need for much prior knowledge and observation data, slow calculation speed	Deep Neural Networks [18] Big Data with Probability Function [19] Sequential Monte Carlo Methods [40] K-Nearest Neighbor Classifier [41]
Other methods	Advanced technology, high accuracy	High technical and economic requirements	Drone-Enabled Participation [6] Random Walk Robot Participation [42,43] Hybrid Optimization Algorithm [44,45,46,47]

**Table 3:** Solar radiation intensity classification.

Cloud Condition	Solar Radiation Angle *α*		
	*α* > 60°	35°< *α* ≤ 60°	15° < *α* ≤ 35°
Cloud cover 4/8, or thin clouds at high altitude	Strong	Medium	Weak
Cloud cover 5/8–7/8, cloud height 2134–4877 m	Medium	Weak	Weak
Cloud cover 5/8–7/8, cloud height lower than 2134 m	Weak	Weak	Weak

**Table 4:** Atmospheric stability classification.

Wind Speed	Under Sunshine			Without Sunshine	
	Strong	Medium	Weak	Cloud Cover ≥4/8	Cloud Cover ≤3/8
0–2	A	A–B	B	F	F
2–3	A–B	B	C	E	F
3–4	B	B–C	C	D	E
4–6	C	C–D	D	D	D
>6	D	D	D	D	D

**Table 5:** Pasquill–Gifford diffusion coefficient equation.

Stability Level	$\sigma_{y}\left(m\right)$	$\sigma_{z}\left(m\right)$
A–B	$0.32x{\left(1+0.0004x\right)}^{-1/2}$	$0.24x{\left(1+0.0001x\right)}^{-1/2}$
C	$0.22x{\left(1+0.0004x\right)}^{-1/2}$	0.20x
D	$0.16x{\left(1+0.0004x\right)}^{-1/2}$	$0.14x{\left(1+0.0003x\right)}^{-1/2}$
E–F	$0.11x{\left(1+0.0004x\right)}^{-1/2}$	$0.08x{\left(1+0.0015x\right)}^{-1/2}$

**Table 6:** Details of the six representative standard test functions.

Function Name	Function Equation	Range	Optimal Solution and Value
Sphere	$f(x)=\sum_{i=1}^{n}x_{i}{}^{2}$	$\left|x_{i}\right|\leq 100$	$0,x_{i}=0$
Rosenbrock	$f(x)=\sum_{i=1}^{n-1}\left[100{\left(x_{i+1}-x_{i}{}^{2}\right)}^{2}+{\left(1-x_{i}\right)}^{2}\right]$	$\left|x_{i}\right|\leq 50$	$0,x_{i}=1$
Rastrigin	$f(x)=\sum_{i=1}^{n}\left[x_{i}{}^{2}-10\cos\left(2\pi x_{i}\right)+10\right]$	$\left|x_{i}\right|\leq 50$	$0,x_{i}=0$
Griewank	$f(x)=\frac{1}{4000}\sum_{i=1}^{n}x_{i}{}^{2}-\prod_{i=1}^{n}\cos\left(\frac{x_{i}}{\sqrt{i}}\right)+1$	$\left|x_{i}\right|\leq 30$	$0,x_{i}=0$
Schaffer	$f(x)=0.5+\frac{\sin^{2}\sqrt{\sum_{i=1}^{n}x_{i}{}^{2}}-0.5}{{\left[1+\frac{1}{1000}{\left(\sum_{i=1}^{n}x_{i}{}^{2}\right)}^{2}\right]}^{2}}$	$\left|x_{i}\right|\leq 100$	$0,x_{i}=0$
Ackley	$f(x)=20+e-20e^{-0.2\sqrt{\frac{1}{n}\sum_{i=1}^{n}x_{i}{}^{2}}}-e^{\frac{1}{n}\sum_{i=1}^{n}\cos\left(2\pi x_{i}\right)}$	$\left|x_{i}\right|\leq 30$	$0,x_{i}=0$

**Table 7:** The parameter settings.

Optimization Algorithm	Parameter	Value
DE/GA	Scaling factor *F*	0.5
	Crossover probability *Cr*	0.5
HHO	*σ_ν_*	1
	*β*	1.5
PS	Initial point	(0, 0, 0)
SA	Initial point	(0, 0, 0)
PSO	Inertia factor	0.3
	$c_{1}$	1
	$c_{2}$	1
Grey Wolf Optimization (GWO) [63]	Convergence constant *a*	[2, 0]
Slap Swarm Algorithm (SSA) [64]	Convergence constant *a*	[2, 0]
Whale Optimization Algorithm (WOA) [65]	Convergence constant *c*_1_	[2, 0]

**Table 8:** Qualitative results table of OMO algorithm under 16 sensors.

Target Parameter	Expected Value	Calculated Value	Relative Error (%)
Time (s)	−	12.743	−
$x_{0}$ (m)	8	8.158	1.971
$y_{0}$ (m)	15	15.020	0.131
*Q* (kg/s)	80	79.045	1.193
Optimal value	0	2.464e-15	−

**Table 9:** Qualitative results table of OMO algorithm under 9 sensors.

Target Parameter	Expected Value	Calculated Value	Relative Error (%)
Time (s)	−	10.304	−
$x_{0}$ (m)	8	8.287	3.593
$y_{0}$ (m)	15	15.444	2.960
*Q* (kg/s)	80	76.909	3.864
Optimal value	0	2.356e-13	−

**Table 10:** Qualitative results table of OMO algorithm under 4 sensors.

Target Parameter	Expected Value	Calculated Value	Relative Error (%)
Time (s)	−	8.644	−
$x_{0}$ (m)	8	10.725	34.068
$y_{0}$ (m)	15	14.190	5.401
*Q* (kg/s)	80	84.612	5.765
Optimal value	0	5.694e-23	−

**Table 11:** Qualitative results table of OMO algorithm with a precision of 1e-10 under 16 sensors.

Target Parameter	Expected Value	Calculated Value	Relative Error (%)
Time (s)	−	6.697	−
$x_{0}$ (m)	8	8.285	3.568
$y_{0}$ (m)	15	15.069	0.459
*Q* (kg/s)	80	79.896	0.131
Optimal value	0	2.029e-10	−

**Table 12:** The relationship between the population number *N* and the optimal value of the objective function calculated by the OMO algorithm.

Population Number *N*	Optimal Value	Time (s)
3	8.450e-6	1.678
5	2.130e-7	2.490
8	9.622e-10	3.712
10	3.253e-10	4.561
15	1.187e-11	6.591
20	7.940e-13	8.680
25	7.228e-15	10.723
30	2.464e-15	12.743
50	2.339e-16	21.753
100	4.073e-17	42.479

**Table 13:** The relationship between the iterations and the optimal value of the objective function calculated by the OMO algorithm.

Iterations	Optimal Value	Time (s)
50	1.384e-10	2.249
100	1.457e-13	4.405
200	3.067e-14	8.623
300	2.464e-15	12.743
400	7.305e-16	16.975
500	4.157e-16	21.311
1000	5.238e-16	43.213

**Table 14:** The partial results of the relationship between the speed control constant and the optimal value of the objective function calculated by the OMO algorithm.

*S_H_*	*S_R_*	Optimal Value
4	6	1.447e-11
23	3	1.690e-11
2	43	2.405e-11
15	2	2.683e-11
1	3	3.602e-11
137	1	3.679e-11
422	440	4.382e-4
709	469	4.384e-4
495	844	4.396e-4
869	42	6.804e-4
865	76	7.124e-4
973	329	7.846e-4

**Table 15:** The OMO algorithm’s results under different SNR.

SNR (dB)	$x_{0}\;(m)$	$y_{0}\;(m)$	*Q* (kg/s)	Optimal Value
0	14.916	12.956	61.406	5.565e-2
0.3	15.173	14.886	54.400	6.340e-2
0.6	11.520	13.130	82.052	3.102e-2
1	12.125	13.927	57.669	2.614e-2
5	11.808	14.294	78.303	1.128e-2
10	9.047	14.618	73.611	3.658e-3
15	9.563	15.179	71.538	1.071e-3
20	7.383	14.727	77.701	3.932e-4
30	8.405	15.271	80.032	8.574e-5
50	8.175	14.977	79.100	4.209e-7
100	7.906	14.848	81.372	3.680e-11

**Table 16:** Comparison of results of different optimization algorithms under 16 sensors.

Target Parameters	OMO	PS	SA	PSO	GA	DE	HHO	GWO	SSA	WOA
Time (s)	12.743	4.910	3.028	2.223	6.049	10.129	12.373	13.196	13.514	13.160
$x_{0}$ (m)	8.158	6.207	5.565	7.410	6.379	7.435	8.528	5.276	6.849	8.779
$y_{0}$ (m)	15.020	14.501	10.905	14.175	13.890	15.028	14.530	14.740	15.026	13.188
Q (kg/s)	79.045	62.406	43.929	83.694	75.824	81.038	76.953	83.620	84.956	76.095
$x_{0}$ relative error (%)	1.971	22.418	30.433	7.379	20.262	7.064	6.606	34.044	14.391	9.741
$y_{0}$ relative error (%)	0.131	3.326	27.297	5.497	7.399	0.186	3.132	1.732	0.171	12.080
Q relative error (%)	1.193	21.993	45.089	4.617	5.220	1.298	3.809	4.525	6.195	4.882
Optimal value	2.464e-15	2.309e-6	9.492e-3	5.625e-8	3.302e-4	3.697e-13	8.531e-7	2.712e-7	6.317e-9	2.434e-5

**Table 17:** Comparison of results of different optimization algorithms under 9 sensors.

Target Parameters	OMO	PS	SA	PSO	GA	DE	HHO	GWO	SSA	WOA
Time (s)	10.304	4.429	2.990	1.638	3.867	8.406	10.263	11.170	11.364	11.083
$x_{0}$ (m)	8.287	4.111	10.667	9.549	5.668	8.854	9.644	1.855	7.312	4.460
$y_{0}$ (m)	15.444	12.990	12.908	13.352	13.661	15.001	15.533	10.150	13.190	12.100
Q (kg/s)	76.909	95.762	52.081	90.098	79.938	85.554	73.722	92.250	83.853	83.342
$x_{0}$ relative error (%)	3.593	48.618	33.335	19.363	29.151	10.675	20.544	76.814	8.598	44.252
$y_{0}$ relative error (%)	2.960	13.400	13.947	10.986	8.926	0.010	3.555	32.332	12.065	19.336
Q relative error (%)	3.864	19.703	34.899	12.622	0.077	6.943	7.847	15.313	4.816	4.178
Optimal value	2.356e-13	9.689e-7	6.450e-4	5.182e-8	6.226e-6	1.459e-11	2.012e-7	4.987e-7	1.960e-8	3.161e-4

**Table 18:** Comparison of results of different optimization algorithms under 4 sensors.

Target Parameters	OMO	PS	SA	PSO	GA	DE	HHO	GWO	SSA	WOA
Time (s)	8.644	3.206	2.954	1.341	3.046	7.219	8.833	9.301	9.536	9.390
$x_{0}$ (m)	10.725	2.677	12.369	2.101	6.126	4.446	10.669	3.628	12.061	4.878
$y_{0}$ (m)	14.190	7.641	15.168	9.720	10.597	9.392	15.471	9.127	8.076	11.870
Q (kg/s)	84.612	127.404	43.551	106.466	100.159	101.978	58.645	80.567	104.305	72.035
$x_{0}$ relative error (%)	34.068	66.536	54.607	73.732	23.427	44.428	33.368	54.653	50.768	39.026
$y_{0}$ relative error (%)	5.401	49.060	1.121	35.200	29.354	37.386	3.137	39.151	46.160	20.864
Q relative error (%)	5.765	59.255	45.562	33.082	25.199	27.472	26.694	0.709	30.382	9.956
Optimal value	5.694e-23	1.401e-11	1.384e-6	5.373e-10	3.371e-10	1.075e-11	9.049e-19	5.915e-12	7.136e-14	2.514e-9
